# FTIR-Based Study of Starch Retrogradation and Protein Structure in Chickpea-Enriched Gluten-Free Bread During Storage

**DOI:** 10.3390/foods15030412

**Published:** 2026-01-23

**Authors:** Petra Lončarić, Marko Jukić, Anca Mihaly Cozmuta, Leonard Gigel Mihaly Cozmuta, Alexandra Maria Uivarasan, Anita Pichler, Mirela Lučan Čolić, Jasmina Lukinac

**Affiliations:** 1 Faculty of Food Technology Osijek, Josip Juraj Strossmayer University of Osijek, F. Kuhaca 18, 31000 Osijek, Croatia; petraloncaric98@gmail.com (P.L.); marko.jukic@ptfos.hr (M.J.); anita.pichler@ptfos.hr (A.P.); mirela.lucan@ptfos.hr (M.L.Č.); 2 Department of Chemistry-Biology, Technical University of Cluj Napoca, 430122 Baia Mare, Romania; ancamihalycozmuta@gmail.com (A.M.C.); mihalyleonard@yahoo.com (L.G.M.C.); uivarasanalexandra@yahoo.com (A.M.U.)

**Keywords:** starch retrogradation, protein secondary structure, FTIR spectroscopy, staling kinetics, crumb firmness, water migration

## Abstract

This study investigated the effect of chickpea flour (CF) on the staling behavior of gluten-free bread (GFB) by comparing a formulation containing 50% rice flour (RF) and 50% CF (CFB) with a control bread based on rice flour supplemented with whey protein (RFB). Bread samples were stored at room temperature for up to 7 days. Changes in color, reflectance, starch and protein structure, specific volume, crumb structure, texture, and staling kinetics were monitored. CFB exhibited a darker and more yellow crumb and crust, with lower reflectance intensity, and showed greater color stability during storage. Fourier-transform infrared (FTIR) spectroscopy revealed higher overall starch crystallinity and more stable relative crystallinity degree (RCD) values in CFB (58.74–59.05%) compared to RFB (46.19–40.52%) throughout storage, indicating early amylose-driven ordering and a more stable molecular organization of starch. Protein secondary structure analysis showed that CFB had a higher β-sheet content (35.05–37.49) than the RFB formulation (30.37–31.16), indicating stronger protein aggregation. In contrast, macroscopic quality parameters showed that CFB had lower specific volume (1.65 vs. 1.93) and porosity (17.17 vs. 21.01 cm^3^/g) than RFB, resulting in higher hardness (15.92 vs. 6.15 N) and accelerated staling kinetics (*k*_corr_) (0.28 vs. 0.14 day^−1^), indicating faster crumb firming despite the observed molecular-level stability. Overall, the results demonstrated that CF contributes to enhanced molecular organization of starch and increased nutritional value of GFB, while its technological performance at the macroscopic level remains formulation-dependent. These findings highlight the need for targeted formulation and process optimization to balance molecular stability with desirable textural properties in CFB.

## 1. Introduction

Bread staling is a significant physical and chemical phenomenon due to bread’s role as a staple food [1,2]. It presents a major problem for bread and bakery products, as it shortens shelf-life and leads to bread waste [3]. Although extensively studied [4,5,6,7], the exact mechanism of the staling process, particularly at the molecular level, remains unclear. Chemical and physical changes during bread storage, such as the loss of characteristic aroma, the loss of crust crispiness and the hardening and drying of the crumb, are considered indicators of bread staling [1]. The most plausible causes of bread staling are starch transformation and moisture redistribution. Upon aging, starch molecules undergo a process called retrogradation in which they shift from an amorphous state to an ordered, crystalline structure [8]. Bread staling begins immediately after baking with amylose retrogradation, during which amylose molecules form new hydrogen bonds and initiate changes that cause the bread’s texture to become more elastic. Subsequently, the moisture content of the bread’s interior decreases, the color changes, and the bread hardens, primarily due to amylopectin retrogradation. This process occurs gradually, as starch molecules slowly form hydrogen bonds and release water molecules [6,9].

Gluten is a protein found in many staple foods in the Western diet and has a unique set of functional properties. Despite this, it has become popular to limit its use in the food industry and to produce gluten-free (GF) products [10]. This is because gluten ingestion has been associated with celiac disease (CD), gluten intolerance, wheat allergy, non-celiac gluten sensitivity, gluten-induced enteropathy, gluten ataxia and dermatitis herpetiformis [11]. For patients with gluten-related disorders, the only available treatment is to completely exclude gluten from their diet. This, combined with the popularity of a Western style diet that is rich in gluten and the concurrent global increase in CD diagnoses, has created a growing consumer demand for GF products [12]. One such product is GFB, which faces both social and scientific challenges compared to conventional gluten-rich bread in terms of quality and acceptability [13]. GFB has various quality defects, such as low specific volume, rough, dry, and crumbly texture, low nutritional value, and a high staling rate. These issues are due to a weak protein network, reduced gas expansion, and pure gas retention during dough leavening [14]. Adding milk proteins or protein isolates to GFB is a common strategy, as they improve crumb structure, texture, shelf life, and nutritional value [15].

This solution, however, raises other problems. First, the incorporation of dairy proteins must be avoided for people who are lactose intolerant [16]. Second, bread containing dairy proteins is no longer suitable for a vegan diet, which excludes all types of animal-derived food [17]. A 2021 World Health Organization (WHO) study states that the European population is shifting toward plant-based diets for health and ethical reasons, demonstrating the growing consumer demand for vegan products [18]. Possible vegan adaptations include legume flours, such as chickpea flour, which contain a good amount of protein, provide beneficial health effects, and improve bread structure and functional properties [19,20]. Vinod et al. [10] state that CF offers many nutritional benefits (rich in proteins, vitamins and dietary fibers), potential health benefits (protection against cancer and cardiovascular diseases, and reduction of glycemic response), and technological benefits (enhances dough stability, consistency and loaf volume).

Methods for tracking bread staling can be categorized as chemical, macroscopic, rheological, microscopic, structural, and molecular. All these methods are considered effective and can be selected based on the facilities and conditions available to producers. However, using a variety of methods can provide better insight into staling kinetics [21]. Most researchers believe that texture analysis is one of the best ways to assess bread staling, as it directly affects overall acceptance and taste-panel results [9,22]. Several studies on staling kinetics have modeled crumb-texture kinetics parameters obtained during storage using the Avrami equation [23,24,25,26,27,28]. In addition to texture-based methods, one of the most common and effective techniques for tracking bread staling is Fourier-transform infrared spectroscopy, as its spectral measurements are based on both physical and chemical parameters [29]. FTIR spectroscopy detects changes in molecular vibrations and provides insight into molecular structure and double-helical organization within crystalline starch regions. Unlike traditional methods that provide important macroscopic data, FTIR emphasizes the degree of crystallization and structural reorganization due to starch retrogradation, which is one of the main indicators of bread staling [30,31,32,33]. Additionally, there is a critical need to explore the protein network present in bread and its relation to quality, such as secondary structure, and very few researchers have applied FTIR measurements for this purpose [34]. The same is true for reflectance spectra of bread. Although there are several research studies conducted to track the staling process of GFB [35,36,37,38], none of them provided a combined staling kinetics analysis through colorimetric measurements (reflectance spectra), a texture-based method, and staling kinetics, together with a detailed analysis of FTIR spectra, to track starch retrogradation and secondary protein structures. Furthermore, several studies have investigated the CF potential in GFB formulations [20,39,40,41,42,43,44,45,46,47,48,49], but none have used a multiscale integrative approach to analyze bread staling through starch retrogradation and secondary protein structures, with an addition of color, volume, texture and crumb-properties analyses.

The aim of this paper was to investigate the technological quality and staling kinetics of vegan gluten-free CFB during storage and to evaluate the technological potential of chickpea flour as a plant-based substitute for dairy proteins used in gluten-free RFB, which served as the control sample. A multiscale analytical approach was used, combining molecular (FTIR) and macroscopic analyses (color, volume, texture), to provide a more comprehensive view of CF’s role in the staling kinetics of GFB. The main hypothesis of this research was that CF affects the staling kinetics of GFB and that FTIR analyses can effectively track starch retrogradation and protein structure changes during GFB storage.

## 2. Materials and Methods

### 2.1. Materials

RF (Nutrigold, Zagreb, Croatia) containing 12.4% moisture, 79.2% carbohydrates, 0% sugar, 0.5% fat, 6.5% protein, <0.01% salt and 0.7% ash, CF (Bio, Karlsruhe, Germany) with 7.3% moisture, 48% carbohydrates, 2.7% sugar, 6.2% fat, 22% protein, 0.03% salt, 13% fiber and 3.0% ash, and whey concentrate (SFD Nutrition, Opole, Poland) containing 5.0% moisture, 9.1% sugar, 6.1% fat, 73% protein, <0.3% salt and 0.7% ash were obtained from a local market. Psyllium husks in powder form (GreeLab, Zagreb, Croatia), Xanthan gum (Nutrimed, Zagreb, Croatia), instant yeast (Saccharomyces cerevisiae; di-go, Istanbul, Turkey), sugar (sucrose), salt and sunflower oil (Žito grupa, Čepin, Croatia) were also purchased from local markets.

### 2.2. Bread Making Procedure

CFB and RFB were prepared according to formulations (Table 1). The formulations were based on a total flour weight of 600 g, corresponding to the ingredient weights listed in Table 1. For dough preparation, dry ingredients were weighed and placed in the mixing bowl. In a separate container, the required amounts of salt, sugar and oil were dissolved in water, and this solution was then added to the mixer. The dough was mixed for 2 min at speed I and then for 6 min at speed II, using a stand mixer (Diosna, D-49074, Dierks & Söhne GmbH, Osnabrück, Germany), and then transferred to a work surface. The dough was divided into four equal portions of 337.5 g each, which were placed in baking tins (dimensions: 15 × 8 × 5 cm). The tins were placed in a proofing chamber (Lievox, XEKPT-08EU-B, Unox, Cadoneghe, Italy) at 30 ± 1 °C and 85 ± 5% relative humidity, for 45 min.

After fermentation, the dough surfaces were scored three times before baking. The oven (Decktop, XEKDT-01EU-S, Unox, Cadoneghe, Italy) was preheated to 230 °C. Baking was carried out as follows: 2 min with steam (the oven’s built-in steam function) at 200 °C, 1 min without steam, then 47 min at 175 °C. All bread baking trials and subsequent post-baking analyses were conducted in three independent batches.

The bread making procedure was the same for CFB and RFB, except for the flour/protein differences shown in Table 1.

According to the data in Table 1 and the protein content of the individual raw materials, the calculated protein content values in the gluten-free bread samples (on a dry-matter basis) were 4.9% rice protein and 6.1% whey protein (total 11.0%) in the RFB sample, and 2.7% rice protein and 9.2% chickpea protein (total 11.9%) in the CFB sample.

### 2.3. Storage of Breads

After baking, the breads were allowed to cool at room temperature for approximately 150 min. They were then individually wrapped in polyethylene foil and stored in plastic bags at room temperature for designated periods of 1, 2, 3 and 7 days.

### 2.4. Bread Analysis

The analysis of 1-, 2-, 3- and 7-day old RFB and CFB included crust- and crumb-color determination, their reflectance spectra, FT-IR analysis, and volume and texture determination.

### 2.5. Colorimetric Analysis

Color measurements of both bread types’ crust and crumb were performed using a YL 4560-3nh non-contact benchtop spectrophotometer (Shenzhen Threenh Technology Co., Ltd., Shenzhen, China) and expressed in the CIEL*a*b* color system, using the following parameters: L*—lightness variable (L* = 100 white, L* = 0 black); a*—intensity of green (−127 < a* < 0) or red (0 < a* < +127); and b*—intensity of blue (−127 < b* < 0) or yellow (0 < b* < +127).

### 2.6. Reflectance Spectra

The raw reflectance spectra of the samples were recorded at 1 nm resolution from 360 to 780 nm, using a UV/VIS Lambda 35 spectrometer (PerkinElmer, Waltham, MA, USA) equipped with a Labsphere RSA-PE-20 integrating sphere.

The reflectance spectra provide a detailed color representation. Different wavelengths correspond to perceptible colors: the color purple manifests at wavelengths of 380–450 nm, blue at 450–480 nm, light blue at 480–500 nm, green at 500–560 nm, yellow at 560–590 nm, orange at 590–620 nm, and red at 620–740 nm. Based on these domains, the color can be evaluated using the seven considered shades. Each shade represents the average reflectance value within its corresponding domain.

### 2.7. FTIR Analysis

FTIR spectra of RFB and CFB bread samples stored for 1, 2, 3, and 7 days were recorded using a PerkinElmer Spectrum BX FT-IR spectrometer (PerkinElmer Ltd., Beaconsfield, UK) equipped with a Bruker Platinum attenuated total reflectance (ATR) accessory with a pressure applicator. The instrument operates in the mid-infrared range of 4000–600 cm^−1^. Spectra were collected directly from the central crumb region of the bread loaves without additional sample preparation, except for the pressure applied to ensure proper contact with the ATR crystal.

All spectra were collected at a nominal resolution of 4 cm^−1^, averaging 40 co-added scans. To minimize the influence of atmospheric CO_2_ and H_2_O, background spectra were recorded under the same room conditions and subtracted from the sample spectra. Absorbance spectra were obtained by determining the ratio of the single-beam sample spectra to the corresponding background spectra.

The raw spectra were pre-processed to remove the characteristic CO_2_ peaks, then smoothed and normalized. Normalization standardized all absorption spectra within the 0–1 range. This process eliminates the effect of varying sample thicknesses on the ATR diamond crystal, which would otherwise result in different absorbance intensity ranges.

For starch, after baseline correction, the 900–1150 cm^−1^ region was deconvoluted using Gaussian curve fitting to estimate the relative areas of crystalline and amorphous fractions, allowing calculation of the RCD [50,51], using MS Excel with built-in Solver functions. The crystalline starch area was calculated as the absorbance ratio of 1047/1020 cm^−1^, and the amorphous area as 1020/995 cm^−1^ [51,52,53]. RCD was calculated as the ratio of the area of crystallinity which occurs at wavenumbers 1047 and 995 cm^−1^ to the sum of all starch molecular structure areas: the amorphous region at 1020 cm^−1^, together with the crystalline area mentioned.

Protein secondary structures were analyzed in the amide I region (1700–1600 cm^−1^). Curve fitting was performed using Gaussian functions in MS Excel with built-in Solver functions to obtain the relative contributions of β-sheet (1700–1682 and 1636–1615 cm^−1^), β-turn (1681–1664 cm^−1^), random coil (1645–1637 cm^−1^), and α-helix (1663–1646 cm^−1^) structures. Relative areas under these deconvoluted peaks were used to quantify the percentage of each secondary structure [51,52,53,54].

All analyses were performed on five replicate spectra per sample, and average values were used for statistical comparisons.

**Figure 1:**
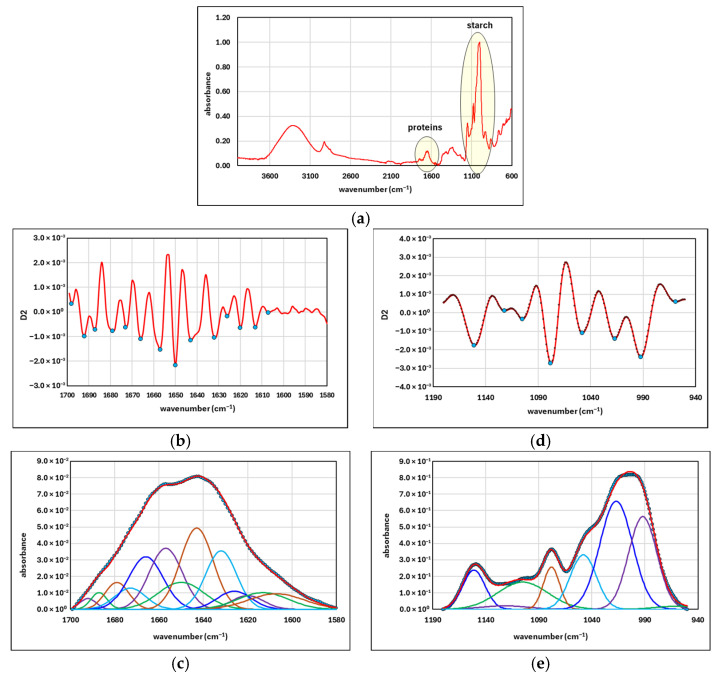
The normalized FTIR spectrum of the analyzed sample (**a**), identification of absorption centers based on second-derivative spectra and the corresponding deconvolution results for determining starch crystallinity degree (**b**,**c**), and the protein secondary structure (**d**,**e**).

The positions of the absorption centers were determined based on the minima of the second-order derivatives, obtained using Savitzky–Golay coefficients. Each position was assigned a Gaussian distribution, initialized with various starting values for the amplification factor and standard deviation. During the deconvolution process, their optimal values were determined so that the sum of the individual distributions overlapped as closely as possible with the FTIR signal over the considered range. Figure 1 shows the normalized FTIR spectrum of an analyzed sample (Figure 1a), the identification of absorption centers and the deconvolution result for determining the starch crystallinity degree (Figure 1b,c), as well as the identification of absorption centers and the deconvolution result for determining the protein secondary structure (Figure 1d,e).

### 2.8. Bread Crumb Structure

Computer-assisted image analysis was used to characterize the crumb morphology of the bread, allowing for an objective assessment of porosity and the distribution of internal cells [55]. Bread slices were digitized with an EPSON Perfection V500 Photo scanner (Seiko Epson Co., Suwa, Nagano, Japan) set to 800 dpi, 24-bit RGB, and saved in TIFF format. The obtained images were processed in ImageJ software (v1.54g, National Institutes of Health, Bethesda, MD, USA), where the central portion of the crumb was defined as the region of interest (ROI), converted to 8-bit grayscale, and subsequently segmented. The resulting binary images were analyzed to obtain the following parameters: cell density (pores/cm^2^), average pore size (mm^2^), and porosity (%).

### 2.9. Volume Analysis

The specific volume of RFB and CFB samples was determined using a Stable Micro Systems VolScan Profiler 300 (Stable Micro Systems Ltd., Surrey, UK). Before analysis, bread samples were removed from polyethylene bags, weighed, and placed in the measuring chamber of the volume scanner.

Volume scanner measurements were performed after entering the sample weight and flour weight for each bread sample. The laser carriage was positioned at 5 mm, and the top pin was used to stabilize the sample during scanning. Scans were conducted in standard resolution mode, with a vertical step of 0.5 mm and a rotation speed of 1 rps.

### 2.10. Texture Analysis

Prior to testing, RFB and CFB samples were removed from polyethylene bags and cut into 2.5 cm-thick slices on days 1, 2, 3, and 7 of storage. For each sample, four slices were analyzed, avoiding the exterior peripheries, with each slice oriented so that the side facing the middle of the loaf was tested.

Texture was measured using a Shimadzu Texture Analyzer EL-LX (Shimadzu Corp., Kyoto, Japan) with an aluminum cylindrical probe (2 cm diameter) in a double-compression test. The probe descended onto the bread surface, compressed the sample (40%), and returned to the surface before performing a second compression. The test speed was set at 2 mm/s. Full-scale settings were CP Force 1000 N and 0.05 N, with break detection enabled. Texture parameters, including hardness, cohesiveness, springiness, adhesive force, chewiness, and resilience, were derived from the resulting force–time curves.

### 2.11. Staling Kinetics

Data for the texture parameters that followed a typical polymer crystallization curve (hardness and chewiness) were analyzed using the Avrami equation to obtain the reaction rate constant *k* and the Avrami exponent *n*. Nonlinear regression was based on the following model [56]:

(1) $$q=\left(X_{L}-X_{t}\right)/\left(X_{L}-X_{0}\right)=exp\left(-k\cdot t^{n}\right),$$

where

*q*—remaining fraction of the parameter change,
*t*—time,
*X*_0_, *X_L_*—initial and limiting experimental value of textural parameter after 1 day and 7 days of storage,
*X_t_*—value at time *t*,
*k*—rate constant,
*n*—Avrami exponent.

For easier comparison of the reaction rate constant *k*, an additional nonlinear regression was carried out with a fixed Avrami exponent (*n* = 1), according to the following model:

(2) $$q=\left(X_{L}-X_{t}\right)/\left(X_{L}-X_{0}\right)=exp\left(-k\cdot t\right)$$

Experimental data for the texture parameters that follow an inverse crystallization trend (springiness, resilience and cohesiveness) were analyzed using the models

(3) $$q=\left(X_{L}-X_{t}\right)/\left(X_{L}-X_{0}\right)=1-exp\left(-k\cdot t^{n}\right)$$

and

(4) $$q=\left(X_{L}-X_{t}\right)/\left(X_{L}-X_{0}\right)=1-exp\left(-k\cdot t\right)$$

### 2.12. Statistical Analysis

All measurements were performed in quintuplicate, and data are reported as mean ± standard deviation (SD). Significant differences (*p* < 0.05) were determined using one-way analysis of variance (ANOVA) followed by Tukey’s multiple comparison test in MS Excel. Staling kinetics for the samples’ texture parameters were analyzed by nonlinear regression using the Statistica software (ver. 14.0.0.15, TIBCO Software Inc., Palo Alto, CA, USA). The obtained staling kinetics parameters were compared using Tukey’s multiple comparison test.

## 3. Results

### 3.1. Color Analysis

Color measurements for both bread types (crust and crumb), expressed in the CIE (International Commission on Illumination) L*a*b* system, are presented in Table 2.

The crust color of CFB differed significantly from RFB in the L* and a* color parameters, with CFB showing a lower L* value and a higher a* value. The mean L* value was 56.7 for RFB and 46.8 for CFB, while the mean a* value was 8.2 for RFB and 12.2 for CFB. The b* color parameter did not differ significantly between bread types, but was slightly lower for CFB (RFB: 30.6, CFB: 28.5). The overall color difference between bread types for the crust was 10.9.

The crumb color of CFB had significantly higher a* (RFB: −5.1, CFB: −2.5) and b* (RFB: 15.5, CFB: 27.6) mean values. The difference in mean L* values between RFB and CFB was not statistically significant (*p* > 0.05) (RFB: 74.4, CFB: 73.6), and the overall color difference between RFB and CFB crumb was 12.4.

### 3.2. Reflectance Spectra

**Figure 2:**
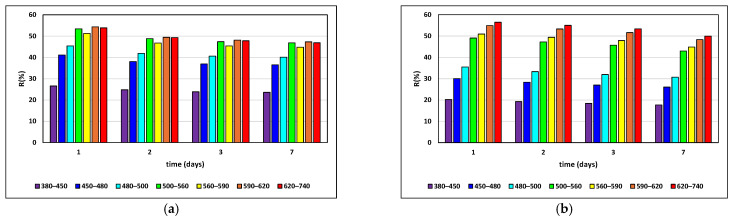

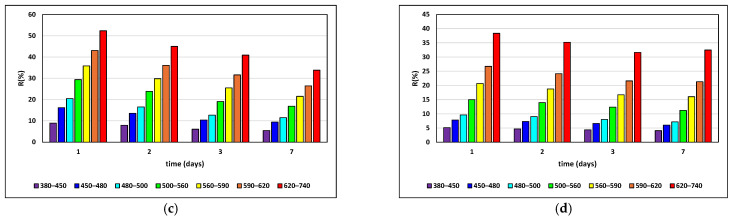
Reflectance spectra (380–740 nm) of rice flour bread (RFB) and chickpea flour bread (CFB) during storage (days 1, 2, 3, and 7): (**a**) RFB crumb; (**b**) CFB crumb; (**c**) RFB crust; (**d**) CFB crust.

The reflectance spectra of RFB and CFB crumb and crust were recorded after 1, 2, 3, and 7 days of storage, and are shown in Figure 2.

The RFB crumb exhibited higher reflectance in the blue and green reflectance regions, while the CFB crumb showed relatively higher reflectance in the red region (Figure 2a,b). For the crust, RFB displayed overall higher reflectance intensity across the visible spectrum compared to CFB (Figure 2c,d). Both bread types showed a gradual and pronounced decrease in reflectance during storage, with changes more intense in the crust than in the crumb. By day 7, reflectance in both RFB and CFB crust and crumb had clearly decreased, but remained comparatively more stable in CFB, especially in the CFB crust.

### 3.3. FTIR Analysis

#### 3.3.1. FTIR Analysis of Starch Structure

FTIR analysis was conducted on days 1, 2, 3, and 7 of storage for both bread types. The evolution of RCD during storage for RFB and CFB is shown in Table 3, while the crystalline (1047/1020) and amorphous regions (1020/995) are presented in Table 4 for both bread types.

CFB exhibited a higher mean RCD value (56.42; Table 3), higher mean crystallinity (0.88), and lower mean amorphous region (1.40) (Table 4) compared to RFB (RCD: 39.24, crystallinity: 0.46, amorphous: 3.75), overall.

During storage, RCD values in both bread types followed a similar trend: decreasing on day 2 (statistically significant only in RFB), slightly increasing on day 3, and increasing further on day 7. RCD values in CFB showed a slight increase between day 1 and day 7 (0.53%), but this change was not statistically significant (*p* > 0.05). In contrast, RCD values in RFB decreased during storage (−12.29%), but this change was also not statistically significant (*p* > 0.05). Neither bread type had statistically significant changes in crystallinity throughout storage overall, although RFB showed a statistically significant (*p* < 0.05) decrease in crystallinity on days 2 and 3 of storage. Additionally, RFB had a statistically significant (*p* < 0.05) decrease in amorphous regions on day 7 of storage, whereas CFB remained stable in this regard (*p* > 0.05).

#### 3.3.2. FTIR Analysis of Secondary Protein Structures

Table 5 shows the evolution of secondary protein structures in RFB and CFB during storage (days 1, 2, 3, and 7), as determined by FTIR analysis.

CFB had higher overall mean values for β-sheet (36.68) and random coil (21.46) secondary protein structures, and lower mean values for α-helix (25.54) and β-turn (16.32) secondary protein structures compared to RFB (α-helix: 31.12, β-sheet: 31.31, β-turn: 17.41, random coil: 20.08). For α-helix content, CFB showed a significant decrease during storage (*p* < 0.05) and a higher percentage change (−15.73%), whereas changes in RFB were not statistically significant and had a lower percentage change (−2.83%).

β-sheet content increased in both bread types (2.59% for RFB, 6.97% for CFB), with a statistically significant increase observed only in CFB (*p* < 0.05). β-turn values showed a statistically significant reduction (*p* < 0.05) for both bread types, larger in CFB (−13.20%) than in RFB (−6.77%).

Random coil content increased significantly in both bread types (*p* < 0.05), with a higher percentage change observed in CFB (20.61%) compared to RFB (6.84%).

### 3.4. Crumb Structure Characteristics

Crumb structure characteristics of RFB and CFB, including cell density, average pore size, porosity, and specific volume, are shown in Table 6.

CFB exhibited a statistically significant higher mean cell density (20.12 pores/cm^3^) compared to RFB (13.32 pores/cm^3^), while showing statistically significant lower mean values for average pore size (0.56 mm^2^), porosity (17.17%) and specific volume (1.65 cm^3^/g) compared to RFB (1.06 mm^2^, 21.01% and 1.93 cm^3^/g, respectively).

### 3.5. Texture Analysis and Staling Kinetics

**Figure 3:**
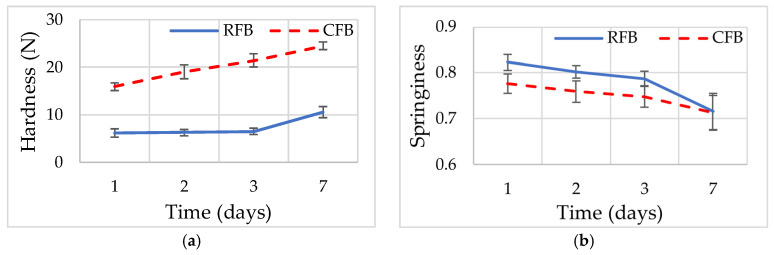

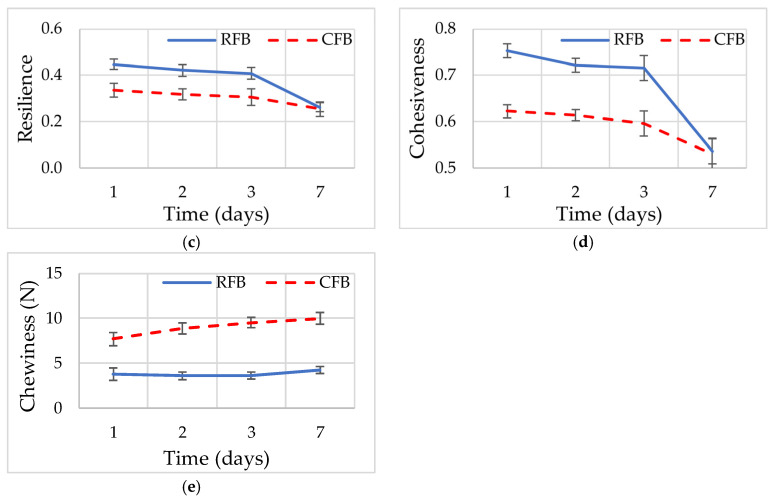
Texture parameters of rice flour bread (RFB) and chickpea flour bread (CFB) on days 1, 2, 3, and 7 of storage: (**a**) hardness; (**b**) springiness; (**c**) resilience; (**d**) cohesiveness; (**e**) chewiness. Error bars represent standard deviation (SD).

Texture parameters including hardness, springiness, resilience, cohesiveness and chewiness, as well as staling kinetics parameters (*X*_0_, *X*_L_, |*X*_L_ − *X*_0_|, *n*, *k*, and *k*_corr_), were determined for RFB and CFB on days 1, 2, 3, and 7 of storage. The results are presented in Table 7 and Figure 3.

Texture profile analysis (TPA) revealed statistically significant differences between RFB and CFB samples during storage. After 1 day of storage, CFB exhibited significantly (*p* < 0.05) higher hardness (15.92 N) and chewiness (7.69 N) than RFB (6.15 N and 3.81 N, respectively). At the same time, springiness, resilience, and cohesiveness were significantly lower in CFB (0.78, 0.33, and 0.62) compared to RFB (0.82, 0.45, and 0.75). During storage, hardness increased significantly in both formulations; however, the increase was significantly greater in CFB (Figure 3a). By day 7, hardness reached 24.46 N in CFB, which was significantly (*p* < 0.05) higher than in RFB (10.51 N). The absolute change in hardness (|*X_L_* − *X*_0_|) was also significantly higher in CFB (8.55 N) than in RFB (4.37 N), consistent with significantly higher kinetic parameters for hardness in CFB (*k* = 0.52 day^−n^; *k*_corr_ = 0.28 day^−1^) compared to RFB (*k* = 0.25 day^−n^; *k*_corr_ = 0.14 day^−1^). Springiness decreased progressively during storage in both breads (Figure 3b). While CFB showed significantly lower springiness than RFB at day 1, no significant differences between formulations were observed at later storage times. Similar trends were observed for resilience and cohesiveness (Figure 3c,d). Both parameters decreased significantly during storage in each formulation; however, differences between RFB and CFB were not statistically significant at day 7 (*p* > 0.05), despite higher absolute reductions in RFB, as indicated by |*X_L_* − *X*_0_| values. Chewiness increased significantly during storage in both breads (Figure 3e), with CFB exhibiting significantly higher values than RFB at all storage times (*p* < 0.05). By day 7, chewiness reached 9.99 N in CFB and 4.28 N in RFB. Consistently, kinetic parameters for chewiness were significantly (*p* < 0.05) higher in CFB (*k* = 0.77 day^−n^; *k*_corr_ = 0.35 day^−1^) than in RFB (*k* = 0.29 day^−n^; *k*_corr_ = 0.26 day^−1^).

Overall, the changes in texture parameters during staling shown in Figure 3 were consistent with the statistical and kinetic analyses presented in Table 7, confirming significantly higher hardening kinetics in CFB, while changes in springiness, resilience, and cohesiveness were more moderate and became statistically comparable between formulations during later storage stages.

## 4. Discussion

Several studies indicate that color is a key factor influencing consumer preferences, and this also applies to bread [57,58].

Compared to conventional wheat bread, GFBs often have a coloration that is inherently too white [13,59,60]. The fact that RFB exhibited significantly different mean values for the crust L* and a* parameters, as well as the a* and b* parameters of the crumb, compared to CFB, is probably due to the color of the flour used in the bread formulation. Many studies have reported that crumb color depends on flour color, which is influenced by grain endosperm color [13,59,60]. Although RF is the most commonly used basic ingredient for the preparation of GFB [61], CF is reported to enhance the sensory properties of bread due to its redness and yellowness [39,62].

According to the results presented in Table 2, CFB crust appears darker and more reddish compared to RFB. Although RFB contains whey protein concentrate, which has been shown to increase the Maillard reaction, and consequently increase color intensity [63,64], the CF used in the GFB formulation also contributes to Maillard reactions. Kahraman et al. [49] conducted research tracking the impact of CF on the technological and nutritional characteristics of GFB, and found that GFBs made with CF had a darker crust than those made with 100% RF, due to the high protein and amino acid content that promotes Maillard browning reactions. Additionally, Atudorei et al. [65] observed that CF contributed to the redness of the crust, which was also seen in the chickpea-containing CFB used in this study. Moreover, Rostamian et al. [40] and Aguilar et al. [41] conducted studies on the influence of CF on GFB, and their results indicated that the darkening of CFB crust was a consequence of Maillard reactions.

The differences in L* values of the crust between bread types could also be due to the different moisture content of RFB and CFB, as the medium with higher moisture content is usually darker than the drier one [66]. CFB may dry more slowly after baking because of the hygroscopic properties of chickpea flour. In this instance, Kahraman et al. [49] observed higher moisture content in all chickpea-containing GFBs compared to GFB containing 100% RF, so the results presented may be due to bread formulation.

A similar pattern can be observed for crumb color between RFB and CFB, with CFB displaying a more yellow shade compared to RFB. These findings are consistent with a study by Santos et al. [46,48], who examined the influence of CF on the sensory properties of bread. They found that the carotenoid pigments in CF imparted a yellowish hue to the experimental CF-based GFB.

The results of the reflectance spectra analysis further support these findings. Figure 3 shows that the RFB crumb had a more defined spectrum in the blue and green color range, while the CFB was more pronounced in the red region. The overall higher intensity displayed by RFB throughout the visible spectrum compared to CFB may be explained by its higher moisture content and the presence of dairy proteins, which promote browning reactions to a greater extent [39,61,64,66]. It should be noted that the control formulation contained whey protein; therefore, the observed differences between RFB and CFB cannot be attributed exclusively to the flour source, but also to differences in protein origin and functionality. Both bread types had a gradual decrease in reflectance, with the effect being more pronounced for the crust than the crumb. The results suggest that the color’s intensity fades over time. Popov-Raljić et al. [66] conducted a study correlating crumb color changes and staling in breads of different compositions packed in polyethylene film for 3 days at 20 °C. The authors attributed the lighter crumb color observed during storage to moisture loss. By fitting the average reflectance values to a curve describing the dependence of average reflectance on storage time, they found a correlation coefficient of 0.99. They concluded that the change in crumb color is a direct consequence of staling. The observed decrease in reflectance *R* (%) during storage in both crumb and crust in our study contrasts with the increase in crumb reflectance reported by Popov and Raljić et al. This discrepancy is primarily due to methodological differences. Popov and Raljić measured reflectance indirectly using the *Y* tristimulus value from a conventional colorimeter, whereas the present study used a spectrophotometer with an integrating sphere to measure total hemispherical reflectance across the spectral range. As a result, the reflectance values reported here are more sensitive to microstructural rearrangements, moisture redistribution, and starch retrogradation during bread staling, rather than to visual lightness alone. Therefore, the opposite trends observed do not represent a contradiction, but instead reflect the fundamentally different optical phenomena captured by the two measurement methods. The results shown in Figure 3 suggest that the changes in RFB were more pronounced. This may be due to more intensive moisture migration from crust to crumb compared to CFB, resulting in more pronounced changes in the reflectance spectrum. While crust staling is strongly influenced by water migration from the crumb to the crust, which substantially modifies surface optical properties, crumb staling is predominantly controlled by amylopectin retrogradation, with moisture migration contributing to a lesser extent [1]. Rosell et al. [67] also stated that water has a crucial role in development of bread color. Although the composition of the gluten-free bread samples analyzed is too heterogeneous (with different protein compositions and varying amounts of rice flour) to draw concrete conclusions, it can be assumed that one reason for the different kinetics of changes during aging in the RFB and CFB samples is the differing amounts and properties of chickpea, whey, and rice proteins (4.9% rice protein and 6.1% whey protein, totaling 11.0%, in the RFB sample; and 2.7% rice protein and 9.2% chickpea protein, totaling 11.9%, in the CFB sample). According to consumer perceptions reported in a study by Santos et al. [45,48], CF and its interaction with psyllium were promising for delaying GFB staling and maintaining acceptability.

The results obtained from FTIR analysis (Table 3 and Table 4) show that CFB had a higher percentage of crystalline area compared to RFB, overall. Both bread types exhibited higher crystallinity and RCD values on day 1 than on day 2 of storage. This can be explained by the formation of crystalline regions immediately after baking due to amylose retrogradation and moisture loss. Demirkesen et al. [68] studied the staling characteristics of GFBs and observed that fresh-bread samples lost moisture more rapidly during the first day of storage. After baking, amylopectin and amylose are in a gelatinized, disordered state, but not all crystallinity is lost. Some residual crystalline regions can remain or re-form during cooling, mostly due to rapid amylose retrogradation [9,69,70,71].

The decrease in RCD between days 1 and 2 of storage for both types of bread should not be interpreted as a reversal of the staling process, but rather as a transient structural reorganization associated with changes in the amorphous fraction. As shown in Table 4, this decrease in RCD coincides with an increase in the amorphous region, suggesting that moisture migration and redistribution within the crumb and from crumb to crust play an important role at this early storage stage. Water, acting as a plasticizer, increases molecular mobility within partially ordered starch regions, leading to a temporary reduction in the relative contribution of crystalline domains to the overall structure. During this period, amylopectin side chains remain mobile at room temperature, and before stable double helices are established, transient associations may rearrange, resulting in a temporary loss of detectable order, rather than a true decrease in crystalline content. Therefore, the observed reduction in RCD most likely reflects a shift in the balance between crystalline and amorphous domains during early-stage staling, rather than a suppression of starch retrogradation. A similar trend of a temporary decrease in relative crystallinity between days 1 and 2 of bread staling was also observed by Ribotta et al. (2004), who used X-ray diffraction (XRD) to monitor crystallization changes [72]. Furthermore, a temporary decrease in relative crystallinity until after day 2 of storage, followed by a further increase, was also reported by Primo-Martín et al. (2007), who conducted research on bread crust using differential scanning calorimetry (DSC) [73]. It should be noted that our FTIR results were not cross-validated with complementary techniques such as DSC or XRD; future studies could include these methods to further corroborate the structural and thermal in-sights obtained from FTIR analysis.

The changes in RCD, along with the decreasing amorphous state and increasing crystalline regions observed in RFB, may be explained by water migrating from the crumb to the crust, indicating that the crumb is losing its softness and the crust is losing its crispiness [9,74,75]. CFB, on the other hand, remained steady across all three parameters used to track the starch retrogradation process—no statistically significant changes were observed in RCD, crystallinity, or amorphous molecular states at any time point. This suggests a more discrete water migration trend. Another possible explanation for this may be the higher amylose content of chickpea starches (30–35% on average) [76,77,78] compared to the average 20–25% in commercial RF, as reported by Suwannaporn et al. (2007) [79]. Throughout the entire storage period, CFB consistently showed a higher overall degree of crystallization than RFB, primarily due to its higher amylose content. Amylose retrogrades rapidly immediately after baking, forming ordered structures during cooling and within the first 24 h of storage [1]. This early and pronounced amylose-driven crystallization establishes a higher baseline level of ordered domains in CFB from the beginning. Consequently, subsequent structural changes associated with amylopectin retrogradation during prolonged storage contribute relatively less to the total crystallinity changes and are therefore less pronounced in relative terms. In this context, the later-stage staling behavior of CFB should not be interpreted as reduced amylopectin involvement, but rather as a reflection of the dominance of early amylose crystallization in determining the overall crystalline framework against which amylopectin reorganization occurs. Furthermore, chickpea has a weaker starch–protein network compared to the gluten-containing cereals, but when compared to RF used in GFB, it may apparently lower the staling rate. Kahraman et al. [49] reported that RFB had the highest staling rate, while CFB had the lowest among the analyzed bread samples. The higher protein and fiber content, as well as lower starch content in CF compared to RF, may have contributed to the lower staling rate, due to their interactions with water [47,74] and starch dilution [80], making the retrogradation process less pronounced. In addition to proteins, other ingredients such as phenols (which are abundant in chickpeas) may influence starch retrogradation through their emulsifying action. Bordenave et al. [81] suggested that flavonoids in particular may interact with starch and thus influence the starch retrogradation process. However, the dairy proteins present in the RFB formulation used in this study may have compensated for these less desirable RF properties. In summary, the data presented in this study suggest that, compared to RFB, CFB exhibited a steadier staling rate and a more resilient starch network, which can be attributed to the CF composition.

The FTIR secondary protein structure analysis further supports the quality of plant-based proteins incorporated into bread formulations. The results show that CFB had a higher content of β-sheet and random coil protein structures, and lower mean values for β-turn and α-helix secondary protein structures compared to RFB. It has been reported that higher β-sheet content promotes the formation of protein networks [82]. Similarly, it can be assumed that higher β-sheet values in CFB, compared to RFB, may result in a more desirable product texture. On the other hand, Zhan et al. [83] report that α-helixes contribute to ordered protein conformations, as this is supported by the results of lower random coil content in RFB. They also observed that an increase in β turn content weakened the protein network, and this protein structure was more prevalent in RFB.

However, during storage, CFB showed a significant decrease in α-helix content, while the decrease in RFB is not statistically significant. The β-sheet is considered the most stable protein conformation because it provides structural stability to the protein network, and its content increased in both bread types, with a greater increase in CFB. β-turn values decreased in both bread types, with a larger reduction in CFB, indicating the resilience of the protein network. As expected, there is a statistically significant increase in random coil structure for both types of bread during storage, indicating the formation of a disordered protein structure [83].

**Figure 4:**
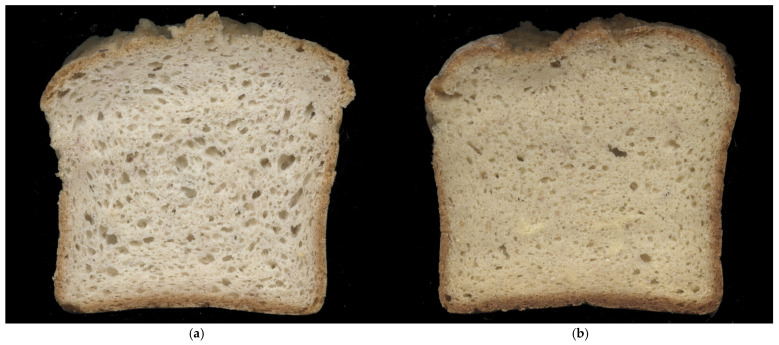
Crumb structure appearance for: (**a**) rice flour bread (RFB); (**b**) chickpea flour bread (CFB).

Cell density represents the number of pores per 1 cm^2^, while porosity is the ratio of the area of gas cells to the area of the bread slice [84]. Specific volume expresses the technological suitability of a formulation for bread production because it reflects loaf crumb structure and gas retention capacity [85,86,87,88,89,90]. Together, these characteristics describe bread crumb structure. In the present study, RFB showed higher mean specific volume, average pore size, and porosity, compared to CFB, as well as lower cell density. This difference is visible to the naked eye (Figure 4).

Since the protein–starch network in bread dough enables gas retention [91], some researchers have proposed that higher specific volume reflects a stronger gluten-like net-work [92]. Taking this into account, these results show a slight contradiction. Krupa-Kozak et al. [93] observed that GFB with 12% milk powders had a significant increase in specific volume, even though RF present in RFB is reported to result in poor specific volume [19]. On the other hand, CF used in CFB formulation has also been reported to increase bread specific volume. Rostamian et al. [40] observed that GFB made with 20% corn flour and 80% CF had a specific volume of 3.55 mL/g by improving porosity. Other researchers [41,42,43,45,46] also concluded that CF raises bread specific volume and improves crumb structure, as a result of its high protein content.

Since these researchers incorporated CF in bread formulations at percentages higher than 50%, or combined it with ingredients other than RF and whey concentrate, it can be assumed that the results obtained here are due to the specific bread formulations used. Bread formulated with RF and CF appears to have a weaker starch–protein network, resulting in higher density and, therefore, lower volume [94,95], compared to the RF + milk protein formulation. Although, as previously noted, the composition of the analyzed GFB samples is too heterogeneous—with varying amounts and sources of proteins and different proportions of rice flour—to allow precise conclusions to be drawn. Higher specific loaf volume results from the foaming capability of bread ingredients and leads to a less cohesive structure that allows greater gas expansion [20]. Although CF has strong foaming capability, its incorporation into bread formulation likely requires further optimization, because the results suggest a greater foaming effect from RFB ingredients (milk proteins).

Texture is one of the most important sensory attributes of bread, making it a key parameter to monitor during storage. By quantifying hardness, springiness, resilience, chewiness, cohesiveness, and adhesive force, it is possible to systematically compare the inevitable and undesirable changes that occur over time [96]. In gluten-containing doughs, these parameters are linked to molecular interactions among the components, including hydrogen bonding, disulfide bridges, cross-linking, and the movement of water within the dough mass [97,98].

CFB, which has higher mean values for hardness and chewiness and lower mean values for springiness, resilience, cohesiveness, and adhesive force, may exhibit these characteristics, due to the higher amylose content of CF, leading to rapid starch retrogradation [9,69,70,71,76,77,78]. Baking conditions and formulation both influence bread texture [99]. Kahraman et al. [49] produced bread with 75% RF and 25% CF, which showed hardness values between 5.5 and 14.1 N. However, Mohammad et al. [100] reported that adding CF to wheat–chickpea blends significantly increased crumb hardness, likely due to thickening of the crumb around the air cells and strengthening of the crumb structure by protein particles. The present results are consistent with those obtained by Lian et al. [44], who found that adding chickpea okara flour to RF-based bread resulted in a 45% increase in crumb hardness. In contrast, Bird et al. [42] observed that adding 2% CF to bread formulation reduced crumb hardness by 40%. These contradictory findings may be due to differences in bread formulations.

Hardness and chewiness were found to follow a typical polymer crystallization curve. The hardness parameter and its evolution during storage are important because they reflect bread softness, and greatly affect consumer impressions [101]. In TPA analysis, hardness represents the force required to compress bread between the molars, which can also be defined as the force necessary to achieve a given deformation [102]. Chewiness represents the energy required to chew bread to the point of swallowing, and is expected to increase during storage [102]. As expected, both bread types showed a significant increase in hardness and chewiness during storage, which is typical for bread stales. The staling kinetics were expressed as the *k*_corr_ value for both bread types. CFB had a significantly higher *k*_corr_ value compared to RFB, suggesting increased crystallization and crystal growth in bread, as assessed by the *n* and *k* parameters of the Avrami equation [28]. Since several researchers have shown that protein content in flour is the main factor influencing the rate of hardening and staling [80,103,104], these results may suggest a steadier staling rate for RFB, due to its different protein network and more stable water migration. Furthermore, chickpea is considered to have a weaker starch–protein network and a higher tendency for starch retrogradation, which can have both positive (better mouthfeel, offsetting the typical dry and crumbly texture of GFB) and negative (risk of rapid staling) effects [44]. The results of the present study are consistent with those of Kotsiou et al. [104], who reported that the staling kinetics of composite breads at a 20% CF substitution level showed greater crumb hardening toward the end of the storage period.

Springiness is associated with freshness. Products with low values are linked to crumb brittleness, and maintaining high springiness values during storage is desirable [105]. Both CFB and RFB showed similar and low *k*_corr_ values for the springiness parameter, suggesting maintenance of freshness and elasticity over time. Substituting RF with CF was shown to increase dough elasticity, resulting in enhanced foaming capacity and stability [43].

Resilience characterizes the initial elasticity of bread, and is calculated as the ratio of the area under the curve of the second half of the first cycle to the first half of the cycle [106]. As predicted from the Avrami parameters derived from springiness values, both bread types exhibited low *k*_corr_ values, with no significant statistical differences.

Cohesiveness indicates the tendency of molecules to remain together in the matrix, and usually decreases during storage as bread loses moisture [102]. This change occurred in both bread types, but overall values were statistically similar. This suggests a more limited water migration trend in both bread types.

The apparent discrepancy between the higher molecular stability observed by FTIR analysis and the faster macroscopic hardening kinetics (*k_corr_*) of CFB underscores the inherently multiscale nature of bread staling. Molecular-level indicators, such as changes in starch and protein secondary structures, primarily reflect local ordering phenomena within the polymer matrix, while macroscopic texture development is determined by higher-order structural features, including pore architecture, cell wall integrity, and mesoscale water distribution. In CFB formulations, early amylose-driven crystallization establishes a relatively stable molecular framework, as observed by FTIR; however, this does not necessarily prevent macroscopic setting, which can be accelerated by reduced gas cell elasticity, denser crumb structure, and altered protein–starch interactions affecting crumb load-bearing elements. Consequently, molecular stability does not directly translate into delayed textural firming, indicating that FTIR-derived parameters and firming kinetics describe complementary, but not directly interchangeable, aspects of staling.

These findings emphasize the importance of formulation- and process-optimization strategies that address both molecular and structural scales. Although CF contributes to early molecular stabilization, its impact on macroscopic texture suggests that additional interventions—such as controlled hydration, optimization of particle size distribution, incorporation of hydrocolloids or enzymes, and adjustment of baking profiles—may be necessary to mitigate crumb firming during storage. The combined use of multiscale analytical tools, as demonstrated in this study, provides a valuable framework to guide scalable formulation strategies aimed at improving the shelf life and textural stability of gluten-free bread products.

## 5. Conclusions

The molecular and textural characteristics of GFB were significantly affected by the addition of CF. FTIR analysis indicated a higher and more stable level of starch organization during storage, likely associated with rapid amylose retrogradation and the compositional characteristics of CF. However, this molecular-level stabilization did not directly translate into reduced macroscopic crumb firming, confirming that bread staling is governed by multiscale mechanisms extending beyond starch retrogradation.

Despite exhibiting a distinct protein network and preserved elastic parameters during storage, CF incorporation negatively affected key technological properties such as specific volume and crumb structure, resulting in accelerated hardening kinetics. These findings highlight the formulation-dependent nature of CFB and emphasize the need for process- and formulation-optimization strategies that address both molecular stability and structural development.

Overall, CF represents a nutritionally valuable, plant-based ingredient with promising functional potential in GFB formulations. The multiscale analytical approach applied in this study provides a useful framework for developing formulation strategies and future research aimed at improving the shelf life and quality of CFB.

## Figures and Tables

**Table 1:** Ingredients and baker’s percentages for RFB and CFB formulations.

Ingredients	Baker’s Percentage—RFB	Baker’s Percentage—CFB
Rice flour	90	50
Chickpea flour	-	50
Whey concentrate	10	-
Xanthan gum	2	2
Psyllium	5	5
Instant yeast	3	3
Oil	5	5
Sugar	3	3
Salt	2	2
Water	105	105

RFB—rice flour bread; CFB—chickpea flour bread.

**Table 2:** Color parameters (L*, a*, b*) and overall color difference (Δ*E*) of crumb and crust of RFB and CFB.

Sample		L*	a*	b*	Δ*E*
Crust	RFB	56.7 ± 3.6 ^a^	8.2 ± 1.1 ^b^	30.6 ± 1.7 ^a^	10.9 ± 1.2
	CFB	46.8 ± 2.4 ^b^	12.2 ± 1.1 ^a^	28.5 ± 1.3 ^a^	
Crumb	RFB	74.4 ± 1.8 ^a^	−5.1 ± 0.1 ^b^	15.5 ± 0.6 ^b^	12.4 ± 0.4
	CFB	73.6 ± 1.6 ^a^	−2.5 ± 0.2 ^a^	27.6 ± 0.7 ^a^	

RFB—rice flour bread; CFB—chickpea flour bread. Values are mean ± SD; superscript letters (a,b) indicate significant differences between bread types within each color parameter (*p* < 0.05, Tukey’s test). Δ*E* represents the overall color difference between RFB and CFB for crust or crumb, calculated from L*, a*, and b* values.

**Table 3:** RCD (%) of RFB and CFB through storage.

Storage (Days)	RFB	CFB
1	46.19 ± 3.13 ^a^	58.74 ± 2.85 ^a^
2	35.04 ± 1.16 ^b^	53.30 ± 3.81 ^a^
3	35.20 ± 1.15 ^b^	54.60 ± 1.46 ^a^
7	40.52 ± 4.24 ^ab^	59.05 ± 4.25 ^a^

RCD—relative crystallinity degree (%); RFB—rice flour bread; CFB—chickpea flour bread. Values are mean ± SD; superscript letters (a,b) indicate significant differences (*p* < 0.05, Tukey’s test) within each bread type.

**Table 4:** Crystallinity and amorphous regions of RFB and CFB during storage.

Parameter	Storage (Days)	RFB	CFB
Crystallinity (1047/1020)	1	0.46 ± 0.05 ^ab^	0.79 ± 0.13 ^a^
	2	0.36 ± 0.08 ^b^	0.73 ± 0.19 ^a^
	3	0.33 ± 0.02 ^b^	0.72 ± 0.11 ^a^
	7	0.68 ± 0.12 ^a^	1.29 ± 0.25 ^a^
Amorphous (1020/995)	1	2.54 ± 0.36 ^ab^	1.29 ± 0.24 ^a^
	2	6.07 ± 0.04 ^a^	1.44 ± 0.33 ^a^
	3	4.89 ± 0.2 ^ab^	1.40 ± 0.20 ^a^
	7	1.49 ± 0.26 ^b^	1.45 ± 0.29 ^a^

RFB—rice flour bread; CFB—chickpea flour bread. Values are mean ± SD; superscript letters (a,b) indicate significant differences (*p* < 0.05, Tukey’s test) within each bread type.

**Table 5:** Secondary protein structure of RFB and CFB during storage.

Secondary Protein Structure	Storage (Days)	RFB	CFB
α-helix	1	31.73 ± 0.29 ^a^	27.18 ± 0.36 ^a^
	2	30.62 ± 0.23 ^a^	25.18 ± 0.34 ^b^
	3	31.30 ± 0.09 ^a^	26.88 ± 0.28 ^a^
	7	30.83 ± 0.87 ^a^	22.91 ± 0.71 ^c^
β-sheet	1	30.37 ± 0.43 ^b^	35.05 ± 0.38 ^b^
	2	32.30 ± 0.26 ^a^	38.36 ± 0.30 ^a^
	3	31.41 ± 0.50 ^ab^	35.81 ± 0.08 ^b^
	7	31.16 ± 1.29 ^ab^	37.49 ± 1.14 ^a^
β-turn	1	18.24 ± 0.18 ^a^	17.61 ± 0.36 ^a^
	2	16.95 ± 0.32 ^b^	15.87 ± 0.26 ^bc^
	3	17.79 ± 0.08 ^a^	16.53 ± 0.52 ^b^
	7	17.01 ± 0.13 ^b^	15.28 ± 0.32 ^c^
Random coil	1	19.66 ± 0.18 ^b^	20.16 ± 0.44 ^b^
	2	20.13 ± 0.30 ^ab^	20.59 ± 0.12 ^b^
	3	19.50 ± 0.37 ^b^	20.78 ± 0.42 ^b^
	7	21.01 ± 0.55 ^a^	24.32 ± 2.00 ^a^

RFB—rice flour bread; CFB—chickpea flour bread. Values are mean ± SD; superscript letters (a,b,c) indicate significant differences (*p* < 0.05, Tukey’s test) within each bread type.

**Table 6:** Crumb structure characteristics of RFB and CFB.

Sample	Cell Density (Pores/cm^2^)	Average Pore Size (mm^2^)	Porosity (%)	Specific Volume (cm^3^/g)
RFB	13.32 ± 2.91 ^b^	1.06 ± 0.15 ^a^	21.01 ± 2.84 ^a^	1.93 ± 0.03 ^a^
CFB	20.12 ± 2.21 ^a^	0.56 ± 0.06 ^b^	17.17 ± 1.13 ^b^	1.65 ± 0.03 ^b^

RFB—rice flour bread; CFB—chickpea flour bread. Values are mean ± SD; superscript letters (a,b) indicate significant differences between bread types within each parameter (*p* <0.05, Tukey’s test).

**Table 7:** Staling kinetics of RFB and CFB.

Parameter	Sample	Hardness (N)	Springiness	Resilience	Cohesiveness	Chewiness (N)	Parameter
*X*_0_	RFB	6.15 ± 0.91 ^b^	0.82 ± 0.02 ^a^	0.45 ± 0.02 ^a^	0.75 ± 0.01 ^a^	3.81 ± 0.67 ^b^	6.15 ± 0.91 ^b^
	CFB	15.92 ± 0.80 ^a^	0.78 ± 0.02 ^b^	0.33 ± 0.03 ^b^	0.62 ± 0.01 ^b^	7.69 ± 0.76 ^a^	15.92 ± 0.80 ^a^
*X*_L_	RFB	10.51 ± 1.17 ^b^	0.72 ± 0.04 ^a^	0.26 ± 0.02 ^a^	0.54 ± 0.03 ^a^	4.28 ± 0.37 ^b^	10.51 ± 1.17 ^b^
	CFB	24.46 ± 0.80 ^a^	0.71 ± 0.04 ^a^	0.25 ± 0.03 ^a^	0.53 ± 0.03 ^a^	9.99 ± 0.62 ^a^	24.46 ± 0.80 ^a^
|*X*_L_ − *X*_0_|	RFB	4.37 ± 0.58 ^b^	0.11 ± 0.02 ^a^	0.18 ± 0.01 ^a^	0.22 ± 0.02 ^a^	0.47 ± 0.39 ^b^	4.37 ± 0.58 ^b^
	CFB	8.55 ± 0.07 ^a^	0.06 ± 0.02 ^b^	0.08 ± 0.00 ^b^	0.09 ± 0.02 ^b^	2.30 ± 0.41 ^a^	8.55 ± 0.07 ^a^
*n*	RFB	1.48 ± 0.04 ^b^	1.47 ± 0.02 ^a^	1.44 ± 0.02 ^a^	1.43 ± 0.02 ^a^	1.60 ± 0.13 ^b^	1.48 ± 0.04 ^b^
	CFB	1.71 ± 0.16 ^a^	1.58 ± 0.22 ^a^	1.50 ± 0.09 ^a^	1.48 ± 0.06 ^a^	2.18 ± 0.31 ^a^	1.71 ± 0.16 ^a^
*k* (day^−n^)	RFB	0.25 ± 0.02 ^b^	0.33 ± 0.03 ^a^	0.28 ± 0.01 ^a^	0.27 ± 0.01 ^a^	0.29 ± 0.08 ^b^	0.25 ± 0.02 ^b^
	CFB	0.52 ± 0.10 ^a^	0.42 ± 0.15 ^a^	0.35 ± 0.09 ^a^	0.30 ± 0.04 ^a^	0.77 ± 0.15 ^a^	0.52 ± 0.10 ^a^
*R* ^2^	RFB	0.885	0.927	0.868	0.847	0.919	0.885
	CFB	0.992	0.953	0.905	0.872	0.986	0.992
*k_corr_* (day^−1^)	RFB	0.14 ± 0.02 ^b^	0.20 ± 0.02 ^a^	0.17 ± 0.01 ^a^	0.16 ± 0.01 ^a^	0.26 ± 0.01 ^b^	0.14 ± 0.02 ^b^
	CFB	0.28 ± 0.03 ^a^	0.23 ± 0.06 ^a^	0.20 ± 0.05 ^a^	0.17 ± 0.02 ^a^	0.35 ± 0.04 ^a^	0.28 ± 0.03 ^a^

RFB—rice flour bread; CFB—chickpea flour bread. Values are mean ± SD; superscript letters (a,b) indicate significant differences (*p* < 0.05, Tukey’s test) within each bread type; *X*_0_, *X_L_*—initial and limiting experimental value of textural parameter after 1 day and 7 days of storage; *n*—Avrami exponent; *k*—rate constant; *k*_corr_—corrected rate constant (fixed Avrami exponent *n* = 1).

## Data Availability

The data that support the findings of this study are available from the corresponding author, Jasmina Lukinac (ptfosptfos2@gmail.com), upon reasonable request.

## Abbreviations

The following abbreviations are used in this manuscript:

ANOVA	Analysis of variance
ATR	Attenuated total reflectance
CD	Celiac disease
CF	Chickpea flour
CFB	Chickpea flour bread
CIE	International Commission on Illumination
DSC	Differential Scanning Calorimetry
FTIR	Fourier-transform infrared spectroscopy
GF	Gluten-free
GFB	Gluten-free bread
RCD	Relative crystallinity degree
RF	Rice flour
RFB	Rice flour bread
RGB	Red–green–blue
ROI	Region of interest
SD	Standard deviation
TPA	Texture profile analysis
WHO	World Health Organization
XRD	X-ray Diffraction
